# Two Patient Cases Illustrating the Importance of Addressing Physical and Mental Trauma as a Cause of Pain in Refugee Women

**DOI:** 10.3389/fsoc.2020.00012

**Published:** 2020-03-13

**Authors:** Gunilla Brodda Jansen

**Affiliations:** Division of Rehabilitation Medicine, Department of Clinical Sciences, Karolinska Institutet at Danderyd University Hospital, Stockholm, Sweden

**Keywords:** pain, refugee women, torture, rehabilitation, mental health

## Abstract

Refugees, and more so women than men, are often seeking medical care for a wide range of health-related problems, among which persistent pain has long been recognized as a defining feature. When treating refugee women for any condition, pain and its consequences need to be addressed in the rehabilitation efforts. It is therefore essential that health professionals engaged in the care of refugee women are familiar with the physiology of pain mechanisms, including the biopsychosocial model of pain, as well as the best evidence-based practice in managing pain, acute as well as chronic. Persistent or chronic pain not only causes disability and restricted functioning but also produces psychological impairments, compounding the impact on overall personal and social functioning. Yet, the research literature on health regarding refugees is predominantly targeted at mental health problems without specific reference to pain and pain as a significant cause of distress and disability due to migration (pre-and post). Pain as a consequence of torture is also an issue that is under-assessed when refugees are seeking medical aid for pain-related problems. When aiming at treating refugee women with disabling pain, one can use the same intervention methods that are being used for other chronic pain states. Professionals need, however, to be able to work with less written information and in close co-operation with interpreters. Reviews of the rehabilitation literature have noted a lack of scientifically rigorous studies of multicomponent interventions for refugees. Only few studies have evaluated outcomes of pain management, and the quality of the evidence they provide is very low. The small number of randomized controlled trials and the resulting paucity of information means that recommendations amounting to good clinical practice for refugee women with persistent pain are lacking. The aim of this article, illustrated by two patient cases, is to point out important areas that need to be addressed within the health care system in order to improve health care for refugee women. Pain is a common reason for refugee women to seek medical aid, is a costly burden for the society, and decreases quality of life for these women.

## Introduction

The number of refugees and asylum seekers in the Western world has risen dramatically over the past 10 years (Carta et al., 2016; Bertelsen et al., 2018). Upon arrival to a new country, the refugees are seldom screened for any illnesses, but studies have indicated that they experience psychosocial as well as medical issues causing disability. The disability is often caused by experiences from war and armed conflicts in their home country together with the strenuous travel in relation to escape and forced resettlement as well as postmigration challenges (Akhavan et al., 2007).

This growing number of people with diverse ethnic, cultural, and religious backgrounds raises new challenges for health care providers. Both physicians and patients become frustrated because of difficulty understanding and explaining symptoms. Refugee patients, often non-Western migrants, have difficulty explaining symptoms, physical as well as mental, to the medical profession, often clinicians trained in the West. The patients' problems are therefore taken as “somatization” by professionals (Bäärnhielm and Ekblad, 2000; Rohlof et al., 2014; Zander, 2016). Refugees with traumatic experiences, and more so women than men, are seeking medical care for a wide range of health-related problems, among which persistent pain is one of the major features (Sondra, 2013). Refugee women are often seeking medical aid within the primary care setting (Rohlof et al., 2014; Sigvardsdotter et al., 2016; Zander, 2016; Ekblad et al., 2018). The appointment is often carried out with an interpreter since the patients lack knowledge of the language spoken. In this setting, the time for thorough assessment, screening, and somatic investigation is often limited, and women are often diagnosed with an ICD diagnosis within the F cluster (i.e., depression, anxiety, insomnia, or PTSD). If pain is addressed, it is often regarded as musculoskeletal without any further specification or understanding of the underlying cause.

Many women seeking medical aid for pain-related symptoms are therefore sent to a number of examinations in order to objectify their suffering, such as laboratory testing, MR scanning, and even operation. The understanding of the multifaceted symptomatology is thereby lost and diminished to a unimodal problem. Refugee women with persistent pain are therefore at risk of being over- or undertreated regarding medication, MR scanning, and even surgery, which often worsens the pain, causes it to become chronic, and increases disability; see case 1 below.

When treating refugee women, pain and its consequences need to be thoroughly assessed and addressed in order to understand the complexity of the total mental and somatic medical burden the female patient is suffering from. In this assessment, the consequences of trauma and even torture experiences need to be addressed (Ekblad et al., 2018). According to studies from the Red Cross, it is not uncommon for 20–40% of non-clinical samples of refugee groups to report not only having been traumatized by war experiences but also having experienced torture, physical as well as mental (Sigvardsdotter et al., 2016).

The International Association in the Study of Pain, IASP, announced 2019 the year for “Pain in the most vulnerable,” and, during the year, focused on pain in asylum seekers in publications and presentations (Williams and Baird, 2016).

## Pain Treatment and Rehabilitation

Today there are few rehabilitation options for patients with a refugee background. The assessment of and knowledge about pain in this patient group are very scarce. The medical profession has no or very little knowledge about treatment options for patients with traumatic experiences, since the medical curriculum in most Western countries lacks teaching about immigration and traumatic events such as torture and its consequences. Without the specific knowledge of the manifestations of trauma and torture, the medical profession can easily miss the clinical features. Pain rehabilitation of refugee patients must include a thorough assessment and clinical examination regarding trauma and torture. Treatment should thereafter be offered by a multimodal rehabilitation team (Sondra, 2013). It is, however, necessary that this competence in some way can be offered within the primary care facility where women seek help (Amris and Brodda Jansen, 2019).

Pain affects everyday life and limits patients both emotionally and physically, making female patients reduce their abilities to interact in their new environment. It is essential that health professionals engaged in the care of refugee women be familiar with the multifaceted aspects of chronic pain. This includes an understanding of pain physiology and the mechanisms involved in the chronification of an acute pain experience. This also includes an understanding of the biopsychosocial model of pain and competence in communicating it to this patient group (Brodda Jansen et al., 2011). Persistent pain not only causes disability and restricted functioning but also produces psychological impairments, compounding the impact on overall personal and social functioning. According to one study, immigrants use more pain medication than a Swedish population with persistent pain, even if the clinical picture is pointing at a multifactorial cause (Olsen et al., 2007). Whether this is due to patient demands or is a result of the lack of rehabilitation options for the patient group is, however, unclear. The research literature on health regarding refugees is predominantly targeted at mental health problems, without specific reference to pain and pain as a significant cause of distress and disability due to migration. Pain as a consequence of torture is also an issue that is much under-assessed in the diagnosis when female refugees are seeking medical care for pain-related problems (Olsen et al., 2007).

When aiming at treating female refugees with disabling pain, one can use the same intervention methods that are being used for other chronic pain states. Professionals need, however, to be able to work with less written information and work in close co-operation with interpreters. Reviews of the rehabilitation literature have noted a lack of scientifically rigorous studies of multicomponent interventions for refugees. Only a few studies have evaluated outcomes of pain management, and the quality of evidence they provide is very low (Brodda Jansen et al., 2011; Jansen, 2013). Recommendations for which components are crucial and effective in the rehabilitation efforts for this very sensitive and often seriously traumatized patient group are lacking. Patients can go through a number of medical consultations, often in primary care, without addressing traumatic events. These patients, and especially women, continue to seek medical care in the search for a cure for their longstanding pain (Soares and Grossi, 1999; Brodda Jansen et al., 2011; Amris and Brodda Jansen, 2019). This will hinder the women from moving toward an acceptance of the pain and therefore reduce functioning in their everyday life.

Two patient cases below illustrate the importance of knowledge and awareness of trauma as a cause of persistent pain in refugee women when assessing, examining, and treating such women. The cases also illustrate not only that patients can get better help with this knowledge but also what health care and welfare systems can probably make saving regarding costs for MRI scans, numerous doctor's appointments, and disability pensions.

## Case 1

This case is that of a 49-year-old woman originating from Somalia. She experienced multiple traumas during the Somalian civil war. She sought asylum in Sweden in 2000. Before resettling in the Stockholm region, she lived in three other cities in Sweden. Her family members stayed in Somalia or were killed in the war. Upon arrival, there was no medical investigation regarding war experiences and trauma, and she did not tell anyone about her traumatic experiences. She lives alone and works part time. She often sought medical aid within the primary care setting regarding back pain, headache, nausea, and dizziness. In 2005, her medical records report severe chronic pain in the head, neck, arms, shoulders, groin, chest, back, and legs, weakness in her body, and dizziness. There is no documentation regarding any experiences of traumatic events. Over the period 2006–2010, she was referred to a number of medical specialists and examinations by her general practitioner regarding her pain. The pain was now affecting her to the extent that she had to be on sick leave for longer periods. In 2010, she went through inguinal surgery without any effect on the groin pain. After this operation, the pain increased and spread to the left leg and lumbar region. She was sent to have numerous MRI scans, and the results indicated spondylolisthesis and disc herniation as the cause of her pain. She was therefore referred to surgery and, in 2011, she went through spine fusion surgery. After this operation, she was accepted for temporary disability in 2012. According to her medical record, she was increasingly depressed and was prescribed SSRI and anxiolytic medication. She withdrew from friends and social interaction. In 2016, she was referred to a pain rehabilitation unit. She was assessed by a multi-professional team (i.e., pain specialist, physiotherapist, and psychologist). The pain specialist had experience from working with traumatized and tortured refugees for a specialized unit in Denmark and, during the clinical examination, revealed that the women had been both severely sexually and physically abused in her country of origin.

### Ethical Approval

The Swedish Ethical Review Authority has been informed, and approved verbal consent has been obtained from the Division of Rehabilitation Medicine, Department of Clinical Sciences, Karolinska Institutet.

## Case 2

This is the case of a 42-year-old woman originating from Iraq who had war experiences during the war in the 2000s. She sought asylum in Denmark together with her family, her husband and two children, in 2005. She stayed unemployed and was very isolated at home, with little interaction with people other than close family. After a year, she started complaining about intense pain in the stomach, head, shoulders, leg, and chest. Because of her intense chest pain and an accompanying rise in blood pressure, she was taken to the Emergency Department at the hospital and was put under intensive surveillance with ECG for 24 h several times per month. She also sought medical aid at her primary care center because of the pain in her feet, back, shoulders, and head. The general practitioner examined the patient finally and asked questions regarding traumatic experiences. The woman revealed that she had been imprisoned and tortured. She has been beaten on the soles of the feet (falanga), hung by her arms for several hours, and given electricity to her nipples in order to give information to the military regarding her brother, who was a political dissident against the Saddam Hussein regime. She was referred to Dignity, a former RCT in Copenhagen (a Center for Rehabilitation of Traumatized and Tortured Patients) and assessed by a multi-professional team with knowledge of trauma and torture. She went through a 9-month rehabilitation period with a bio-psycho-social focus, not with the aim of pain reduction, but to increase functioning and activity in daily life and reduce disability. After this period, the patient's pain reduced to some extent. Interestingly, she did not report any stomach or headache pain. As a major positive outcome, the woman decreased her visits to the general practitioner, the number of episodes of chest pain decreased, and she did not seek emergency care for them.

### Ethical Approval

The IRB ethics committee of Dignity—Danish Institute Against Torture waived the need for written informed consent and approved the use of verbal informed consent for the publication of this case report.

The two female patient cases above not only illustrate the importance of taking the experience of trauma in general and torture more specifically into consideration when meeting refugee women with pain. The cases also illustrate the lack of knowledge within the healthcare systems and the immediate need for action regarding specialized units for this patient group. This will not only help patients but will also save money and resources that today are becoming more and more scarce.

Patients have given their verbal consent to publish their cases in a scientific peer review paper; written informed consent has not been obtained. The patient cases have previously only been used in lectures for students, and ethical approval has not been required.

## Conclusions

¤ There is a need to improve knowledge about pain in refugee women in the health care system.
¤ Treatment must focus on bio-psycho-social efforts, not only physical or psychiatric.
¤ Education about pain and trauma should be mandatory for students and specialists at all levels.
¤ Specialized units are needed to assess and sometimes treat this patient group.
¤ Teamwork and rehabilitation skills are needed in order to increase function and improve quality of life.

## Author Contributions

The author confirms being the sole contributor of this work and has approved it for publication.

### Conflict of Interest

The author declares that the research was conducted in the absence of any commercial or financial relationships that could be construed as a potential conflict of interest.
